# 1-Benzyl-3-(2-furylmeth­yl)-1,2,3,4,5,6-hexa­hydro­spiro­[benzo[*h*]quinazoline-5,1′-cyclo­hexa­ne]-2,4-dione

**DOI:** 10.1107/S1600536808007502

**Published:** 2008-03-29

**Authors:** Rafael Tamazyan, Armen Ayvazyan, Ashot Markosyan, Siranush Gabrielyan

**Affiliations:** a Molecular Structure Research Center, National Academy of Sciences RA, Azatutyan ave. 26, 375014 Yerevan, Republic of Armenia; b Institute of Fine Organic Chemistry, National Academy of Sciences RA, Azatutyan ave. 26, 375014 Yerevan, Republic of Armenia

## Abstract

The title compound, C_29_H_28_N_2_O_3_, displays anti­depressant and anti­cancer activities. The furan ring is disordered over two orientations [site occupancies 0.690 (12)/0.310 (12)] related by a rotation of 180°. The ring conformations are chair for the cyclo­hexane ring, boat for the cyclo­hexa­diene ring and twist for the pyrimidine ring. The crystal packing is determined solely by van der Waals inter­actions.

## Related literature

For the synthesis and biological properties of related compounds, see: Markosyan *et al.* (1991 ▶, 1995 ▶). For reference structural data, see: Markosyan *et al.* (1999 ▶, 2000 ▶). For related literature, see: Kuroyan *et al.* (1989 ▶).
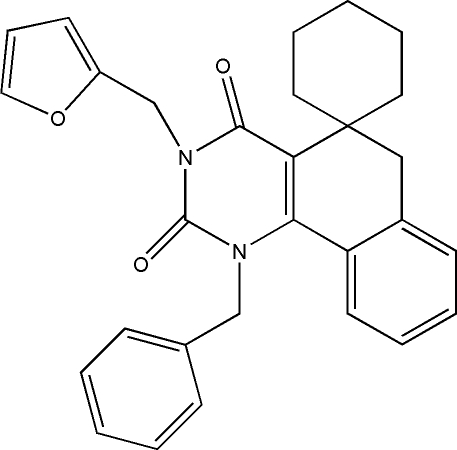


## Experimental

### 

#### Crystal data

C_29_H_28_N_2_O_3_
*M* *_r_* = 452.53
Triclinic, 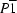

*a* = 10.615 (2) Å
*b* = 11.472 (2) Å
*c* = 11.923 (2) Å
α = 109.90 (2)°
β = 97.95 (2)°
γ = 115.41 (2)°
*V* = 1162.0 (5) Å^3^
*Z* = 2
Mo *K*α radiation
μ = 0.08 mm^−1^
*T* = 293 (2) K
0.42 × 0.4 × 0.3 mm

#### Data collection

Enraf–Nonius CAD-4 diffractometer
Absorption correction: none
7089 measured reflections
6747 independent reflections
4438 reflections with *I* > 2σ(*I*)
*R*_int_ = 0.012
3 standard reflections frequency: 180 min intensity decay: none

#### Refinement

*R*[*F* ^2^ > 2σ(*F* ^2^)] = 0.060
*wR*(*F* ^2^) = 0.148
*S* = 1.14
6747 reflections
444 parameters
13 restraints
H atoms treated by a mixture of independent and constrained refinement
Δρ_max_ = 0.20 e Å^−3^
Δρ_min_ = −0.19 e Å^−3^

### 

Data collection: *CAD-4 Software* (Enraf–Nonius, 1988 ▶); cell refinement: *CAD-4 Software*; data reduction: *HELENA* (Meetsma & Spek, 2000 ▶; program(s) used to solve structure: *SHELXS97* (Sheldrick, 2008 ▶); program(s) used to refine structure: *SHELXL97* (Sheldrick, 2008 ▶); molecular graphics: *SHELXTL* (Sheldrick, 2008 ▶); software used to prepare material for publication: *SHELXTL*.

## Supplementary Material

Crystal structure: contains datablocks global, I. DOI: 10.1107/S1600536808007502/kp2162sup1.cif

Structure factors: contains datablocks I. DOI: 10.1107/S1600536808007502/kp2162Isup2.hkl

Additional supplementary materials:  crystallographic information; 3D view; checkCIF report

## Figures and Tables

**Fig. 1.**
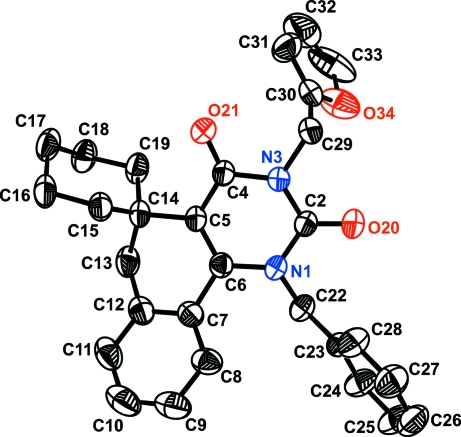
The molecular structure with displacement ellipsoids drawn at the 50% probability level (H atoms omitted for clarity).

**Fig. 2.**
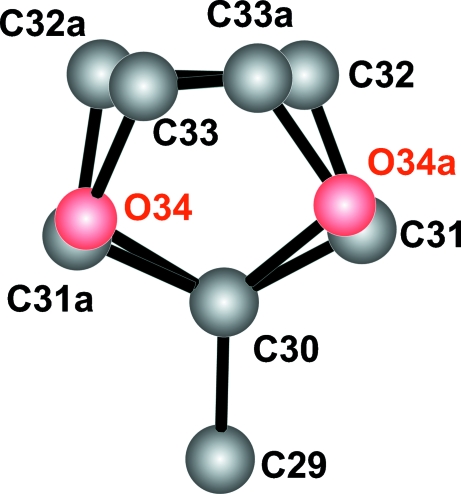
A disorder model of furan ring.

**Fig. 3.**
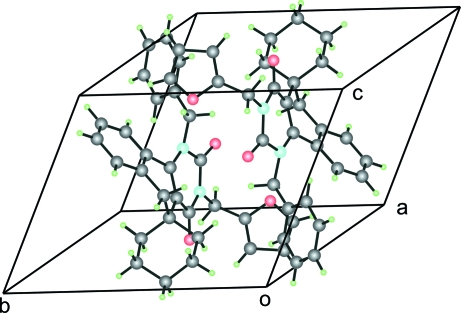
The crystal packing of I via van der Waals interactions, only.

## supplementary crystallographic information

### Comment

The title compound (Fig. 1) shows antidepressant and anticancer activities. All the bond lengths and angles are in good agreement with reference values (Allen *et al.*, 1995). In the crystal structure the two orientations of furan ring are observed in the ratio 0.7 : 0.3, which are related by rotation about C29—C30 bond for 180° (Fig. 2). The crystal packing is realised through van der Waals interactions (Fig. 3).

### Experimental

The title compound was synthesized in the three step reaction: (i) The mixture of 28.5 g (0.1 mol) 4-amino-3-ethoxycarbonyl-1,2- dihydrospiro(naphtalene-2,1'-cyclohexane) (Kuroyan *et al.* 1989) and 15.6 g (0.1 mol) phenylchloroformate in 100 ml dry benzene was boiled with back-flow condenser for 7 h. After solvent distillation the residue phenylester of 3-ethoxicarbonyl-1,2-dihydrospiro(naphtalene-2,1'-cyclohexane) -2-carbaminic acid was recrystallized from mixture ethanol-water (3: 1); (ii) The mixture of 4.0 g (0,01 mol) phenylester of 3-ethoxicarbonyl- 1,2-dihydrospiro(naphtalene-2,1'-cyclohexane)-2-carbaminic acid and 9.71 g (0.01 mol) furan-2-ylmethylamine in 20 ml absolute ethanol was boiled with back-flow condenser for 7 h. To the reaction mixture 1.1 g (0.02 mol) KOH in 10 ml water was added and again boiled for 3 h. The reaction mixture was cooled to 283 K and acidulated by HCl. (iii) The mixture of 1.81 g (0.005 mol) 3-(furan-2-ylmethyl)-1,2,3,4,5,6-hexahydrospiro(benzo[*h*]quinazoline- 5,1'-cyclohexane)-2,4-dione, 0.34 g (0.006 mol) KOH, and 0.64 g (0.005 mol) benzylchloride in 30 ml absolute ethanol was boiled with back-flow condenser for 10 h. After cooling to room temperature 20 ml water was added and the precipitate of 1-benzyl-3-(furan-2-ylmethyl)-1,2,3,4,5,6-hexahydrospiro(benzo[*h*]quinazoline- 5,1'-cyclohexane)-2,4-dione was filtered off and recrystallized from butanole.

### Refinement

The analysis of difference Fourier maps indicated disorder for furan group. Because of disorder of furan ring (C31, C32, C33 and O34) SADI instructions were aplied on corresponding interatomic distances in furan ring. The H atom positions except of those belonging to furan ring were determined from difference Fourier maps and their positions and *U*_iso_ values were freely refined. H atoms of furan group were positioned geometrically and refined using a riding model with C—H = 0.93 Å and *U*_iso_(H) = 1.2*U*_eq_(C).

### Crystal data

**Table:**

C_29_H_28_N_2_O_3_	*Z* = 2
*M**_r_* = 452.53	*F*_000_ = 480
Triclinic, *P*1	*D*_x_ = 1.293 Mg m^−^^3^
Hall symbol: -P 1	Mo *K*α radiation λ = 0.71073 Å
*a* = 10.615 (2) Å	Cell parameters from 22 reflections
*b* = 11.472 (2) Å	θ = 13–16º
*c* = 11.923 (2) Å	µ = 0.08 mm^−^^1^
α = 109.90 (2)º	*T* = 293 (2) K
β = 97.95 (2)º	Prism, colourless
γ = 115.41 (2)º	0.42 × 0.4 × 0.3 mm
*V* = 1162.0 (5) Å^3^	

### Data collection

**Table:**

Enraf–Nonius CAD-4 diffractometer	*R*_int_ = 0.012
Radiation source: fine-focus sealed tube	θ_max_ = 30.0º
Monochromator: graphite	θ_min_ = 1.9º
*T* = 293(2) K	*h* = 0→14
θ/2θ scans	*k* = −16→14
Absorption correction: none	*l* = −16→16
7089 measured reflections	3 standard reflections
6747 independent reflections	every 180 min
4438 reflections with *I* > 2σ(*I*)	intensity decay: none

### Refinement

**Table:**

Refinement on *F*^2^	Secondary atom site location: difference Fourier map
Least-squares matrix: full	Hydrogen site location: difference Fourier map
*R*[*F*^2^ > 2σ(*F*^2^)] = 0.060	H atoms treated by a mixture of independent and constrained refinement
*wR*(*F*^2^) = 0.148	*w* = 1/[σ^2^(*F*_o_^2^) + (0.0526*P*)^2^ + 0.5819*P*] where *P* = (*F*_o_^2^ + 2*F*_c_^2^)/3
*S* = 1.14	(Δ/σ)_max_ < 0.001
6747 reflections	Δρ_max_ = 0.20 e Å^−^^3^
444 parameters	Δρ_min_ = −0.19 e Å^−^^3^
13 restraints	Extinction correction: none
Primary atom site location: structure-invariant direct methods	

### Special details

**Table:**

Geometry. All e.s.d.'s (except the e.s.d. in the dihedral angle between two l.s. planes) are estimated using the full covariance matrix. The cell e.s.d.'s are taken into account individually in the estimation of e.s.d.'s in distances, angles and torsion angles; correlations between e.s.d.'s in cell parameters are only used when they are defined by crystal symmetry. An approximate (isotropic) treatment of cell e.s.d.'s is used for estimating e.s.d.'s involving l.s. planes.
Refinement. Refinement of *F*^2^ against ALL reflections. The weighted *R*-factor *wR* and goodness of fit *S* are based on *F*^2^, conventional *R*-factors *R* are based on *F*, with *F* set to zero for negative *F*^2^. The threshold expression of *F*^2^ > σ(*F*^2^) is used only for calculating *R*-factors(gt) *etc*. and is not relevant to the choice of reflections for refinement. *R*-factors based on *F*^2^ are statistically about twice as large as those based on *F*, and *R*- factors based on ALL data will be even larger.

### Fractional atomic coordinates and isotropic or equivalent isotropic displacement parameters (Å^2^)

**Table:**

	*x*	*y*	*z*	*U*_iso_*/*U*_eq_	Occ. (<1)
N1	1.08120 (15)	0.21666 (16)	0.48300 (13)	0.0419 (3)	
C2	1.13299 (19)	0.14849 (19)	0.39899 (17)	0.0449 (4)	
N3	1.04486 (15)	0.07554 (16)	0.27309 (14)	0.0432 (3)	
C4	0.89672 (18)	0.03776 (19)	0.23022 (17)	0.0420 (4)	
C5	0.83797 (18)	0.08654 (18)	0.32815 (16)	0.0394 (4)	
C6	0.93588 (18)	0.18943 (19)	0.44529 (16)	0.0395 (4)	
C7	0.8947 (2)	0.2859 (2)	0.52898 (16)	0.0444 (4)	
C8	0.9960 (3)	0.4318 (2)	0.60849 (18)	0.0532 (5)	
H8	1.099 (2)	0.470 (2)	0.6160 (19)	0.053 (6)*	
C9	0.9475 (3)	0.5213 (3)	0.6720 (2)	0.0674 (6)	
H9	1.022 (3)	0.625 (3)	0.730 (3)	0.096 (9)*	
C10	0.7987 (4)	0.4663 (3)	0.6557 (2)	0.0754 (8)	
H10	0.763 (3)	0.529 (3)	0.702 (3)	0.094 (9)*	
C11	0.6971 (3)	0.3226 (3)	0.5753 (2)	0.0657 (6)	
H11	0.594 (3)	0.281 (2)	0.560 (2)	0.070 (7)*	
C12	0.7436 (2)	0.2304 (2)	0.51091 (18)	0.0505 (5)	
C13	0.6409 (2)	0.0748 (3)	0.4216 (2)	0.0543 (5)	
H13A	0.539 (3)	0.051 (2)	0.404 (2)	0.063 (6)*	
H13B	0.650 (2)	0.012 (2)	0.458 (2)	0.066 (7)*	
C14	0.67232 (18)	0.0313 (2)	0.29541 (17)	0.0417 (4)	
C15	0.6232 (2)	0.0922 (2)	0.21252 (19)	0.0457 (4)	
H15A	0.670 (2)	0.196 (2)	0.2642 (18)	0.047 (5)*	
H15B	0.662 (2)	0.077 (2)	0.1407 (18)	0.045 (5)*	
C16	0.4546 (2)	0.0179 (3)	0.1570 (2)	0.0569 (5)	
H16A	0.413 (2)	0.041 (2)	0.226 (2)	0.066 (7)*	
H16B	0.433 (2)	0.060 (2)	0.102 (2)	0.061 (6)*	
C17	0.3820 (2)	−0.1444 (3)	0.0866 (2)	0.0623 (6)	
H17A	0.276 (3)	−0.190 (2)	0.052 (2)	0.062 (6)*	
H17B	0.416 (2)	−0.170 (2)	0.012 (2)	0.060 (6)*	
C18	0.4207 (2)	−0.2040 (3)	0.1724 (3)	0.0637 (6)	
H18A	0.377 (3)	−0.311 (3)	0.127 (2)	0.076 (7)*	
H18B	0.379 (3)	−0.187 (2)	0.242 (2)	0.070 (7)*	
C19	0.5887 (2)	−0.1354 (2)	0.2251 (2)	0.0549 (5)	
H19A	0.626 (2)	−0.163 (2)	0.154 (2)	0.063 (7)*	
H19B	0.614 (2)	−0.172 (2)	0.286 (2)	0.069 (7)*	
O20	1.25037 (15)	0.15498 (16)	0.43382 (14)	0.0613 (4)	
O21	0.83070 (15)	−0.02697 (16)	0.11618 (12)	0.0576 (4)	
C22	1.1571 (2)	0.2637 (2)	0.61735 (18)	0.0465 (4)	
H22A	1.172 (2)	0.185 (2)	0.6173 (19)	0.052 (6)*	
H22B	1.088 (2)	0.270 (2)	0.6644 (19)	0.053 (6)*	
C23	1.30282 (19)	0.40402 (19)	0.68204 (16)	0.0432 (4)	
C24	1.3708 (3)	0.4532 (2)	0.80950 (19)	0.0587 (5)	
H24	1.322 (2)	0.397 (2)	0.854 (2)	0.066 (7)*	
C25	1.5049 (3)	0.5808 (3)	0.8754 (2)	0.0734 (7)	
H25	1.544 (3)	0.612 (3)	0.966 (3)	0.101 (9)*	
C26	1.5726 (3)	0.6612 (3)	0.8161 (2)	0.0684 (7)	
H26	1.669 (3)	0.753 (3)	0.865 (2)	0.081 (7)*	
C27	1.5061 (3)	0.6136 (3)	0.6900 (2)	0.0652 (6)	
H27	1.550 (3)	0.667 (3)	0.644 (2)	0.085 (8)*	
C28	1.3712 (2)	0.4848 (2)	0.6228 (2)	0.0562 (5)	
H28	1.325 (3)	0.451 (3)	0.534 (2)	0.074 (7)*	
C29	1.1047 (2)	0.0178 (2)	0.17737 (19)	0.0465 (4)	
H29A	1.079 (2)	0.038 (2)	0.104 (2)	0.061 (6)*	
H29B	1.209 (2)	0.072 (2)	0.217 (2)	0.056 (6)*	
C30	1.0443 (2)	−0.1381 (2)	0.13117 (17)	0.0478 (4)	
C31	0.9483 (10)	−0.2341 (9)	0.0171 (7)	0.088 (3)	0.690 (12)
H31	0.9064	−0.2183	−0.0459	0.105*	0.690 (12)
C32	0.9225 (10)	−0.3754 (10)	0.0129 (10)	0.098 (3)	0.690 (12)
H32	0.8649	−0.4666	−0.0553	0.118*	0.690 (12)
C33	0.9995 (11)	−0.3411 (8)	0.1272 (8)	0.096 (4)	0.690 (12)
H33	0.9968	−0.4091	0.1541	0.116*	0.690 (12)
O34	1.0839 (7)	−0.1958 (6)	0.2025 (5)	0.0702 (19)	0.690 (12)
O34A	0.9428 (10)	−0.2586 (10)	0.0228 (9)	0.069 (4)	0.310 (12)
C31A	1.1165 (16)	−0.1635 (14)	0.2101 (15)	0.043 (3)	0.310 (12)
H31A	1.1921	−0.0967	0.2871	0.052*	0.310 (12)
C33A	0.9426 (18)	−0.3671 (17)	0.046 (2)	0.091 (8)	0.310 (12)
H33A	0.8724	−0.4638	−0.0058	0.109*	0.310 (12)
C32A	1.0492 (16)	−0.3246 (14)	0.1478 (17)	0.061 (3)	0.310 (12)
H32A	1.0763	−0.3804	0.1746	0.073*	0.310 (12)

### Atomic displacement parameters (Å^2^)

**Table:**

	*U*^11^	*U*^22^	*U*^33^	*U*^12^	*U*^13^	*U*^23^
N1	0.0344 (7)	0.0459 (8)	0.0370 (7)	0.0204 (6)	0.0044 (6)	0.0127 (6)
C2	0.0342 (8)	0.0422 (9)	0.0465 (10)	0.0186 (7)	0.0052 (7)	0.0119 (8)
N3	0.0328 (7)	0.0456 (8)	0.0415 (8)	0.0208 (6)	0.0086 (6)	0.0098 (6)
C4	0.0336 (8)	0.0414 (9)	0.0445 (9)	0.0200 (7)	0.0071 (7)	0.0133 (8)
C5	0.0321 (8)	0.0429 (9)	0.0429 (9)	0.0211 (7)	0.0095 (7)	0.0174 (7)
C6	0.0386 (8)	0.0425 (9)	0.0400 (9)	0.0225 (7)	0.0122 (7)	0.0193 (7)
C7	0.0543 (10)	0.0537 (11)	0.0363 (9)	0.0344 (9)	0.0166 (8)	0.0223 (8)
C8	0.0695 (14)	0.0562 (12)	0.0409 (10)	0.0380 (11)	0.0195 (9)	0.0211 (9)
C9	0.101 (2)	0.0645 (15)	0.0490 (12)	0.0547 (15)	0.0256 (13)	0.0226 (11)
C10	0.115 (2)	0.095 (2)	0.0566 (14)	0.0813 (19)	0.0412 (15)	0.0354 (14)
C11	0.0774 (16)	0.102 (2)	0.0561 (13)	0.0665 (16)	0.0361 (12)	0.0431 (14)
C12	0.0579 (11)	0.0719 (13)	0.0422 (10)	0.0430 (11)	0.0236 (9)	0.0315 (10)
C13	0.0416 (10)	0.0761 (15)	0.0568 (12)	0.0321 (10)	0.0223 (9)	0.0369 (11)
C14	0.0306 (8)	0.0490 (10)	0.0476 (10)	0.0218 (7)	0.0120 (7)	0.0225 (8)
C15	0.0406 (9)	0.0538 (11)	0.0487 (10)	0.0279 (9)	0.0146 (8)	0.0245 (9)
C16	0.0431 (10)	0.0814 (16)	0.0610 (13)	0.0392 (11)	0.0172 (9)	0.0376 (12)
C17	0.0341 (10)	0.0795 (16)	0.0625 (14)	0.0240 (10)	0.0091 (9)	0.0298 (12)
C18	0.0361 (10)	0.0613 (14)	0.0780 (16)	0.0150 (10)	0.0098 (10)	0.0311 (12)
C19	0.0364 (9)	0.0485 (11)	0.0710 (14)	0.0184 (9)	0.0077 (9)	0.0257 (10)
O20	0.0404 (7)	0.0691 (9)	0.0610 (9)	0.0328 (7)	0.0034 (6)	0.0136 (7)
O21	0.0463 (7)	0.0715 (9)	0.0397 (7)	0.0336 (7)	0.0043 (6)	0.0082 (6)
C22	0.0413 (10)	0.0501 (11)	0.0410 (9)	0.0202 (9)	0.0054 (8)	0.0201 (8)
C23	0.0417 (9)	0.0427 (9)	0.0380 (9)	0.0222 (8)	0.0055 (7)	0.0127 (7)
C24	0.0644 (13)	0.0550 (12)	0.0400 (10)	0.0236 (10)	0.0025 (9)	0.0185 (9)
C25	0.0755 (16)	0.0595 (14)	0.0471 (12)	0.0223 (12)	−0.0109 (11)	0.0117 (11)
C26	0.0519 (12)	0.0483 (12)	0.0713 (15)	0.0160 (10)	−0.0048 (11)	0.0138 (11)
C27	0.0569 (13)	0.0543 (13)	0.0678 (14)	0.0181 (11)	0.0167 (11)	0.0248 (11)
C28	0.0536 (12)	0.0540 (12)	0.0439 (11)	0.0179 (10)	0.0086 (9)	0.0196 (9)
C29	0.0356 (9)	0.0496 (11)	0.0483 (10)	0.0223 (8)	0.0151 (8)	0.0144 (9)
C30	0.0433 (10)	0.0529 (11)	0.0508 (11)	0.0299 (9)	0.0204 (8)	0.0181 (9)
C31	0.116 (6)	0.060 (3)	0.081 (5)	0.046 (4)	0.023 (4)	0.028 (3)
C32	0.098 (6)	0.048 (3)	0.104 (5)	0.017 (3)	0.050 (5)	0.008 (3)
C33	0.146 (10)	0.101 (6)	0.145 (8)	0.096 (7)	0.116 (8)	0.095 (6)
O34	0.110 (4)	0.067 (3)	0.062 (3)	0.055 (3)	0.048 (2)	0.039 (3)
O34A	0.036 (3)	0.041 (5)	0.079 (6)	0.021 (3)	−0.024 (4)	−0.014 (4)
C31A	0.041 (4)	0.029 (4)	0.044 (5)	0.012 (3)	0.010 (3)	0.010 (3)
C33A	0.045 (5)	0.044 (6)	0.130 (16)	0.028 (5)	−0.011 (8)	−0.011 (8)
C32A	0.053 (5)	0.047 (5)	0.091 (7)	0.033 (4)	0.043 (5)	0.022 (5)

### Geometric parameters (Å, °)

**Table:**

N1—C2	1.380 (2)	C18—H18A	1.00 (3)
N1—C6	1.406 (2)	C18—H18B	1.00 (2)
N1—C22	1.479 (2)	C19—H19A	1.00 (2)
C2—O20	1.219 (2)	C19—H19B	1.01 (2)
C2—N3	1.383 (2)	C22—C23	1.505 (3)
N3—C4	1.401 (2)	C22—H22A	0.98 (2)
N3—C29	1.480 (2)	C22—H22B	0.99 (2)
C4—O21	1.222 (2)	C23—C28	1.376 (3)
C4—C5	1.457 (2)	C23—C24	1.383 (3)
C5—C6	1.359 (2)	C24—C25	1.380 (3)
C5—C14	1.527 (2)	C24—H24	0.99 (2)
C6—C7	1.482 (2)	C25—C26	1.373 (4)
C7—C8	1.397 (3)	C25—H25	0.97 (3)
C7—C12	1.400 (3)	C26—C27	1.369 (3)
C8—C9	1.388 (3)	C26—H26	1.00 (3)
C8—H8	0.96 (2)	C27—C28	1.392 (3)
C9—C10	1.380 (4)	C27—H27	0.97 (3)
C9—H9	1.00 (3)	C28—H28	0.97 (2)
C10—C11	1.382 (4)	C29—C30	1.471 (3)
C10—H10	1.00 (3)	C29—H29A	1.01 (2)
C11—C12	1.397 (3)	C29—H29B	0.95 (2)
C11—H11	0.95 (2)	C30—C31A	1.317 (6)
C12—C13	1.491 (3)	C30—C31	1.317 (6)
C13—C14	1.548 (3)	C30—O34	1.363 (4)
C13—H13A	0.96 (2)	C30—O34A	1.364 (4)
C13—H13B	0.99 (2)	C31—C32	1.505 (8)
C14—C15	1.541 (3)	C31—H31	0.9300
C14—C19	1.547 (3)	C32—C33	1.316 (6)
C15—C16	1.532 (3)	C32—H32	0.9300
C15—H15A	0.98 (2)	C33—O34	1.365 (4)
C15—H15B	0.99 (2)	C33—H33	0.9300
C16—C17	1.513 (4)	O34A—C33A	1.365 (4)
C16—H16A	1.00 (2)	C31A—C32A	1.505 (8)
C16—H16B	0.99 (2)	C31A—H31A	0.9300
C17—C18	1.511 (3)	C33A—C32A	1.316 (6)
C17—H17A	0.97 (2)	C33A—H33A	0.9300
C17—H17B	1.00 (2)	C32A—H32A	0.9300
C18—C19	1.531 (3)		
			
C2—N1—C6	121.11 (14)	C19—C18—H18A	107.3 (14)
C2—N1—C22	114.30 (15)	C17—C18—H18B	109.7 (14)
C6—N1—C22	119.42 (15)	C19—C18—H18B	111.1 (13)
O20—C2—N1	121.86 (17)	H18A—C18—H18B	106.0 (19)
O20—C2—N3	122.46 (17)	C18—C19—C14	113.22 (18)
N1—C2—N3	115.66 (15)	C18—C19—H19A	109.4 (13)
C2—N3—C4	124.67 (15)	C14—C19—H19A	107.7 (13)
C2—N3—C29	117.84 (14)	C18—C19—H19B	109.9 (13)
C4—N3—C29	117.07 (15)	C14—C19—H19B	108.3 (13)
O21—C4—N3	118.32 (16)	H19A—C19—H19B	108.2 (18)
O21—C4—C5	126.01 (16)	N1—C22—C23	116.35 (16)
N3—C4—C5	115.61 (15)	N1—C22—H22A	104.9 (12)
C6—C5—C4	117.92 (15)	C23—C22—H22A	109.7 (12)
C6—C5—C14	121.80 (16)	N1—C22—H22B	107.8 (12)
C4—C5—C14	120.10 (15)	C23—C22—H22B	109.3 (12)
C5—C6—N1	121.11 (16)	H22A—C22—H22B	108.6 (17)
C5—C6—C7	119.36 (15)	C28—C23—C24	118.73 (19)
N1—C6—C7	119.14 (16)	C28—C23—C22	124.40 (17)
C8—C7—C12	120.01 (18)	C24—C23—C22	116.87 (18)
C8—C7—C6	122.61 (18)	C25—C24—C23	120.3 (2)
C12—C7—C6	116.64 (17)	C25—C24—H24	120.1 (13)
C9—C8—C7	120.1 (2)	C23—C24—H24	119.6 (14)
C9—C8—H8	120.7 (13)	C26—C25—C24	120.8 (2)
C7—C8—H8	119.2 (12)	C26—C25—H25	122.8 (17)
C10—C9—C8	119.9 (3)	C24—C25—H25	116.4 (17)
C10—C9—H9	121.0 (16)	C27—C26—C25	119.4 (2)
C8—C9—H9	119.0 (17)	C27—C26—H26	120.6 (15)
C9—C10—C11	120.5 (2)	C25—C26—H26	120.0 (15)
C9—C10—H10	120.7 (16)	C26—C27—C28	120.2 (2)
C11—C10—H10	118.9 (16)	C26—C27—H27	122.2 (16)
C10—C11—C12	120.6 (2)	C28—C27—H27	117.7 (16)
C10—C11—H11	123.3 (15)	C23—C28—C27	120.6 (2)
C12—C11—H11	116.1 (15)	C23—C28—H28	119.3 (14)
C11—C12—C7	118.9 (2)	C27—C28—H28	120.0 (14)
C11—C12—C13	123.8 (2)	C30—C29—N3	112.69 (16)
C7—C12—C13	117.29 (17)	C30—C29—H29A	109.4 (12)
C12—C13—C14	112.36 (17)	N3—C29—H29A	107.8 (12)
C12—C13—H13A	111.2 (13)	C30—C29—H29B	112.1 (13)
C14—C13—H13A	109.3 (13)	N3—C29—H29B	105.6 (13)
C12—C13—H13B	111.5 (13)	H29A—C29—H29B	109.0 (17)
C14—C13—H13B	106.2 (13)	C31A—C30—C31	125.7 (9)
H13A—C13—H13B	106.0 (18)	C31—C30—O34	114.2 (5)
C5—C14—C15	111.34 (15)	O34—C30—O34A	101.9 (6)
C5—C14—C19	107.97 (15)	C31A—C30—C29	110.5 (6)
C15—C14—C19	110.40 (16)	C31—C30—C29	123.0 (5)
C5—C14—C13	107.42 (15)	O34—C30—C29	122.9 (3)
C15—C14—C13	111.66 (16)	O34A—C30—C29	135.2 (6)
C19—C14—C13	107.89 (17)	C30—C31—C32	103.6 (7)
C16—C15—C14	112.83 (17)	C30—C31—H31	128.2
C16—C15—H15A	112.2 (11)	C32—C31—H31	128.2
C14—C15—H15A	107.6 (11)	C33—C32—C31	104.7 (8)
C16—C15—H15B	107.7 (11)	C33—C32—H32	127.7
C14—C15—H15B	108.5 (11)	C31—C32—H32	127.7
H15A—C15—H15B	107.9 (15)	C32—C33—O34	113.3 (7)
C17—C16—C15	111.99 (18)	C32—C33—H33	123.4
C17—C16—H16A	108.3 (13)	O34—C33—H33	123.4
C15—C16—H16A	109.8 (13)	C30—O34—C33	103.9 (4)
C17—C16—H16B	112.1 (12)	C30—O34A—C33A	102.7 (10)
C15—C16—H16B	107.4 (12)	C30—C31A—C32A	104.6 (10)
H16A—C16—H16B	107.2 (18)	C30—C31A—H31A	127.7
C18—C17—C16	110.7 (2)	C32A—C31A—H31A	127.7
C18—C17—H17A	110.2 (13)	C32A—C33A—O34A	115.0 (15)
C16—C17—H17A	110.9 (13)	C32A—C33A—H33A	122.5
C18—C17—H17B	109.5 (13)	O34A—C33A—H33A	122.5
C16—C17—H17B	109.6 (13)	C33A—C32A—C31A	102.8 (15)
H17A—C17—H17B	105.7 (18)	C33A—C32A—H32A	128.6
C17—C18—C19	110.35 (19)	C31A—C32A—H32A	128.6
C17—C18—H18A	112.3 (14)		
			
C6—N1—C2—O20	−169.53 (18)	C5—C14—C15—C16	169.10 (17)
C22—N1—C2—O20	−15.1 (3)	C19—C14—C15—C16	49.2 (2)
C6—N1—C2—N3	11.9 (3)	C13—C14—C15—C16	−70.8 (2)
C22—N1—C2—N3	166.29 (16)	C14—C15—C16—C17	−53.5 (2)
O20—C2—N3—C4	165.43 (19)	C15—C16—C17—C18	57.6 (3)
N1—C2—N3—C4	−16.0 (3)	C16—C17—C18—C19	−58.2 (3)
O20—C2—N3—C29	−6.9 (3)	C17—C18—C19—C14	56.0 (3)
N1—C2—N3—C29	171.69 (16)	C5—C14—C19—C18	−172.93 (19)
C2—N3—C4—O21	−179.91 (18)	C15—C14—C19—C18	−51.0 (3)
C29—N3—C4—O21	−7.5 (3)	C13—C14—C19—C18	71.3 (2)
C2—N3—C4—C5	2.8 (3)	C2—N1—C22—C23	81.6 (2)
C29—N3—C4—C5	175.20 (16)	C6—N1—C22—C23	−123.49 (18)
O21—C4—C5—C6	−162.17 (19)	N1—C22—C23—C28	−3.5 (3)
N3—C4—C5—C6	14.9 (2)	N1—C22—C23—C24	176.44 (18)
O21—C4—C5—C14	13.0 (3)	C28—C23—C24—C25	−0.3 (3)
N3—C4—C5—C14	−169.97 (15)	C22—C23—C24—C25	179.7 (2)
C4—C5—C6—N1	−19.1 (3)	C23—C24—C25—C26	0.4 (4)
C14—C5—C6—N1	165.81 (16)	C24—C25—C26—C27	−0.3 (4)
C4—C5—C6—C7	153.65 (16)	C25—C26—C27—C28	0.1 (4)
C14—C5—C6—C7	−21.4 (3)	C24—C23—C28—C27	0.0 (3)
C2—N1—C6—C5	5.5 (3)	C22—C23—C28—C27	180.0 (2)
C22—N1—C6—C5	−147.66 (17)	C26—C27—C28—C23	0.1 (4)
C2—N1—C6—C7	−167.29 (16)	C2—N3—C29—C30	101.6 (2)
C22—N1—C6—C7	39.6 (2)	C4—N3—C29—C30	−71.3 (2)
C5—C6—C7—C8	−142.40 (19)	N3—C29—C30—C31A	−82.3 (10)
N1—C6—C7—C8	30.5 (3)	N3—C29—C30—C31	107.7 (7)
C5—C6—C7—C12	27.8 (2)	N3—C29—C30—O34	−72.8 (4)
N1—C6—C7—C12	−159.32 (16)	N3—C29—C30—O34A	104.9 (8)
C12—C7—C8—C9	1.5 (3)	C31A—C30—C31—C32	10.5 (15)
C6—C7—C8—C9	171.33 (18)	O34—C30—C31—C32	−0.6 (10)
C7—C8—C9—C10	−0.5 (3)	O34A—C30—C31—C32	−10 (4)
C8—C9—C10—C11	−0.8 (4)	C29—C30—C31—C32	178.9 (5)
C9—C10—C11—C12	1.1 (4)	C30—C31—C32—C33	3.9 (11)
C10—C11—C12—C7	−0.1 (3)	C31—C32—C33—O34	−6.0 (11)
C10—C11—C12—C13	−179.3 (2)	C31A—C30—O34—C33	−145 (5)
C8—C7—C12—C11	−1.2 (3)	C31—C30—O34—C33	−2.8 (9)
C6—C7—C12—C11	−171.64 (17)	O34A—C30—O34—C33	−0.7 (8)
C8—C7—C12—C13	178.10 (18)	C29—C30—O34—C33	177.7 (4)
C6—C7—C12—C13	7.7 (2)	C32—C33—O34—C30	5.6 (10)
C11—C12—C13—C14	132.1 (2)	C31A—C30—O34A—C33A	7.5 (16)
C7—C12—C13—C14	−47.2 (2)	C31—C30—O34A—C33A	169 (5)
C6—C5—C14—C15	105.2 (2)	O34—C30—O34A—C33A	−1.9 (13)
C4—C5—C14—C15	−69.8 (2)	C29—C30—O34A—C33A	−179.9 (11)
C6—C5—C14—C19	−133.43 (19)	C31—C30—C31A—C32A	−7(2)
C4—C5—C14—C19	51.6 (2)	O34—C30—C31A—C32A	36 (4)
C6—C5—C14—C13	−17.3 (2)	O34A—C30—C31A—C32A	−2.5 (18)
C4—C5—C14—C13	167.71 (17)	C29—C30—C31A—C32A	−177.0 (10)
C12—C13—C14—C5	49.9 (2)	C30—O34A—C33A—C32A	−10 (2)
C12—C13—C14—C15	−72.5 (2)	O34A—C33A—C32A—C31A	9(3)
C12—C13—C14—C19	166.05 (16)	C30—C31A—C32A—C33A	−4(2)
